# Case of Amputation at the Instep

**Published:** 1816-11

**Authors:** John C. Yeatman

**Affiliations:** Member of the Royal College of Surgeons, &c.


*For the London Medical and Physical Journal.*

### *Case of Amputation at the Instep;* by JOHN C. YEATMAN, Member of the Royal College of Surgeons, &c.

DULY impressed with the important advantages resulting to the public from all improvements in surgery, especially those in which human suffering is most deeply concerned, I have been lately induced to perform an ope-

 ration ration on the foot analogous to that recommended by Chopart; and, as I have succeeded therein to my complete satisfaction, I take this method of giving it publicity.

My attention to the subject was solely directed by an extract from Villermé's Memoir, which appeared in your Journal for January,* an extract, which, as it seems to contain the substance of that valuable document, and as I have neither seen Chopart's description of his operation or the memoir, I make no apology for quoting in this communication.

"The following memoir, on partial Amputation of the Foot, by L. R. Villermé, D. M. P. contains an account of Chopart's operation, first published in 1792, which has for its object preserving the posterior part of the foot, by amputating at such articulations of the tarsus as circumstances admit of. The operation was followed by a partial reunion, from the first intention, of the two flaps which were formed, to the articulating surfaces of the bone, and complete cicatrization occurred at the end of a month by suppuration of the rest of the wound. Chopart deserves much credit for this operation. A most important part is preserved, by which both standing and walking are secured to the subject of incurable ulceration of the other part of the feet, and which, before this improvement, had always been most strikingly impeded by the usual operation, viz. amputation of the lower leg."

"Chopart tells us, so successful is this treatment, that the patient stands in no need of mechanical assistance afterwards, but can walk perfectly well without it. This, however, was not supported by the experience of Villermé: he says he was led to believe, at the first examination, if we could add to the remaining extremity of the foot another mechanical extremity, thus augmenting the base of support which has been diminished by the operation, we shall effect the cure of the disease, and supply the loss from amputation; for we thus replace in some measure, or form anew, the portion of the dismembered extremity. This artificial extremity will have all the perfection of which it is susceptible, if maintained in the direction of the foot by a strong interior spring."

"It will be impossible, in this article, more than to mention the advantages Mr. Villermé urges in favour of the operation he recommends, or to point out in what it differs from that of Chopart. The author is astonished that the process of Chopart should not have led to new trials. It was not till 1812 that notice was taken of it by M. Richerand,

---

* See vol. xxxv. p. 74.

in in the Nosographe Chirurgicale, and in the Memoirs of Military Surgery, by Mr. Larrey. The advantages proposed by Villermé appear to depend more particularly, 1st, on the remaining length of that portion of the foot which is anterior to its articulation with the leg; and on its size, which both better insure walking, and prevent falling forward: 2d, on the facility we shall have in supplying an artificial extremity to the foot, equal to the whole extent of the base of support, and the whole length of the column which forms the inferior limb."—Ibid.

I shall now enter, without circumlocution, upon the nature and treatment of the case in point, and, after making one or two observations, leave facts to speak for themselves.

Sarah, daughter of James Slade, Morgan's-lane, Frome, æt. four, was brought to me with gangrenous toes of the left foot, occurring, without inflammation, after low fever. The mother of the child stated, that a few days ago she perceived a black spot at the end of one of the little toes, which came on during the night, without pain, swelling, or redness of the foot, and which spread very rapidly.

Sept. 28th.—*Local appearances.* All the toes, and a small portion of the foot, of a jet-black colour, cold, and devoid of feeling; a slough of the size of a shilling under the heel, and, moving it a little aside with the nail of my fore-finger, a point of the os calcis to be seen denuded and black.

*General symptoms.* Face rather flushed, tongue slightly furred, pulse quick and small, bowels loose, appetite bad.

Having placed the child's leg and foot upon a soft pillow, midway between flexion and extension, I ordered a beer-grounds fermenting poultice, the P. e cretâ cum opio, and a nourishing diet.

30th.—A line of separation forming around the foot at the middle of the metatarsal bones. General symptoms improving. Poultice continued, powders omitted, and small doses of bark with opiate confection prescribed.

April 2d.—Sloughs baring the anterior half of the metatarsus and the whole of the first phalanges. Healthy granulations forming. Poultice and bark continued.

6th.—No symptom of irritation apparent; appetite tolerably good. Bark discontinued. The new surface dressed with digestive ointment under the poultice.

8th.—Sore under the heel nearly well. From this time to the 18th, I waited for the complete restoration of healthy action in those soft parts whence sloughs had arisen, and, conceiving that now to be the case, proceeded to amputate at the junction of the tarsus and metatarsus. Having dissected the integuments, with the remaining portions of the extensor extensor and flexor tendons, and plantaris muscle, sufficiently high for my purpose, my pupil holding them back, I removed the five ossa metatarsa, by dividing their lateral, capsular, and other ligaments, which bind them strongly to the bones of the tarsus, and which, to avoid the appearance of anatomical display, I omit mentioning. The external plantar artery (the only vessel that bled) secured, the plantaris muscle formed a sufficient cushion to the stump, and the skin, readily brought together by slips of adhesive, healed by the first intention, excepting a small point over the os cuboides, through which the ligature passed. The stump became perfectly sound in a fortnight, and the child enabled to walk, barefooted, without difficulty, though certainly not without some *fear of falling forward.*

To remedy this inconvenience, I have, besides directing the upper leather of the child's left quarter boot to be of a softer and thinner texture than that of its fellow, ordered the space between the stump and toe to be filled with cork, convex towards the stump, and room enough left for a layer of wool to be insinuated between them; and also a steel plate to be included between the inner and outer sole, within the stitches and channel, and extending from the heel to the toe, the plate being gradually thinner as it approaches the latter point. With this very cheap and simple contrivance, the base of support to the inferior limb is augmented, and sufficient elasticity given to the foot to enable the child to walk and run *without the least hesitation or visible deformity.*

Cases of the foregoing kind have been permitted to form their own stumps. Now, there are two great objections to this practice, 1st, that Nature's stump is without a cushion; and 2d, that the new-formed parts with which she covers it, are, by a well-known law in physiology, liable to ulcerate. In the above case, however, I must not omit stating that the metatarsal bones retained their periosteum for certain distances only beyond their articulations with the tarsus: the toes, therefore, if left to nature, would have separated at their *first phalanges,* and *portions* of the *metatarsus* must have exfoliated, laying the foundation of abscesses, or sinusses, greatly impairing the child's constitution, and leaving her with a weak and mutilated stump.

In regard to Chopart's operation, the author of the memoir before noticed naturally expresses his surprise that it has not been adopted by others; and, indeed, it is not, perhaps, a little extraordinary, that, while we admire the ingenuity and merit of one of our own countrymen in reviving and improving the apparently ludicrous operation of Taliacotius, so wittily described by the inimitable Butler, that this improvement in modern surgery is neglected, after being successfully tried by a skilful Frenchman, noticed by Richerand and Parrey, and extolled by Villermé,—for, although the repairing a man's *nose* may be highly *ornamental*, yet the preserving his leg, and half his foot, cannot be less important.

*Frome; Sept.* 1816.

