# Corrigenda

**Published:** 1816-12

**Authors:** 


					Corrigenda
-At page 358, line 19 from the top, for convcx read
concave ; p. 359, 1. 4, for Parrey read Lurrey ;

				

