# Errata

**Published:** 1817-06

**Authors:** 


### ERRATA.

Vol. 36, page 358, line 19, for *convex,* read *concave.*—Page 359, line 4, for *Parrey* read *Larrey.*

